# Carson’s Synopsis

**Published:** 1868-02-01

**Authors:** 


***Carson's Synopsis of***

Materia Medica, and Bouchardat's Annual Abstract for 1867, received too late for notice in the present No.

